# A Non-Obese Hyperglycemic Mouse Model that Develops after Birth with Low Birthweight

**DOI:** 10.3390/biomedicines10071642

**Published:** 2022-07-08

**Authors:** Daichi Katayama, Nobuhiko Nagano, Shoichi Shimizu, Kimitaka Nakazaki, Kengo Matsuda, Wataru Tokunaga, Kazumasa Fuwa, Ryoji Aoki, Ichiro Morioka

**Affiliations:** Department of Pediatrics and Child Health, Nihon University School of Medicine, Tokyo 173-8610, Japan; katayama.daichi@nihon-u.ac.jp (D.K.); nagano.nobuhiko@nihon-u.ac.jp (N.N.); shimizu.shoichi@nihon-u.ac.jp (S.S.); nakazaki.kimitaka@nihon-u.ac.jp (K.N.); matsuda.kengo@nihon-u.ac.jp (K.M.); tokunaga.wataru@nihon-u.ac.jp (W.T.); fuwa.kazumasa@nihon-u.ac.jp (K.F.); aoki.ryoji@nihon-u.ac.jp (R.A.)

**Keywords:** body composition, developmental origins of health and disease, homeostasis model assessment of insulin resistance, immunoreactive insulin, metabolite analyses, myogenic insulin resistance

## Abstract

The number of low birthweight (LBW) infants weighing below 2500 g has not decreased in Japan. This study aimed to develop an adult non-obese hyperglycemic mouse model born with LBW to study the pathogenesis. At 16.5 days of gestation, transient intrauterine ischemia (blocked blood flow in both uterine arteries for 15 min) was performed in a subgroup of pregnant mice (group I). Non-occluded dams were used as sham controls (group C). After birth, female pups in each group were weaned at 4 weeks of age and reared on the normal diet until 8 weeks of age (*n* = 7). Fasting blood glucose levels, serum immunoreactive insulin (IRI), and body composition were then measured. Metabolite analyses was performed on the liver tissues. Birthweight was significantly lower in group I compared with group C. Pups from group I remained underweight with low fat-free mass and showed hyperglycemia with high serum IRI and homeostasis model assessment of insulin resistance levels, indicating insulin resistance. Metabolite analyses showed significantly reduced adenosine triphosphate and nicotinamide adenine dinucleotide production and increased lactic acid in group I. The pathogenesis of our non-obese hyperglycemic mouse model may be due to increased myogenic insulin resistance based on mitochondrial dysfunction and reduced lean body mass.

## 1. Introduction

Fetuses exposed to undernutrition by lean pregnant women with nutritional restrictions during pregnancy lose weight and acquire insulin resistance and a frugal constitution that easily accumulates energy-efficient fat through the adaptation of metabolic and endocrine mechanisms to the undernutrition environment in utero [1,2]. Low birthweight (LBW) infants are more likely to develop metabolic syndrome and lifestyle-related diseases such as type 2 diabetes mellitus, hyperlipidemia, and hypertension in adulthood (developmental origins of health and disease [DOHaD] theory) [3].

The total number of births in Japan is decreasing, yet the trend of LBW infants weighing under 2500 g has not decreased [4]. The percentage of LBW infants in the total annual number of births is 9.49%, which is higher than that of other countries (8.02% in the United States, 6.95% in the United Kingdom, 6.65% in Germany, 4.95% in China, and 8.38% in Brazil) [5]. Therefore, it is very important to reduce adulthood health problems in LBW infants for medical, economic, and social reasons.

It has been reported that a Japanese patient born with LBW developed type 2 diabetes mellitus without being markedly obese at a young age [6]. Some diabetic patients in Japan are non-obese and have a normal body mass index (BMI, 24.9 kg/m^2^ or less) [7]. Indeed, Japanese type 2 diabetes patients were less obese than those in Western countries [8]. Approximately 15% of Japanese children with type 2 diabetes are non-obese, which is a higher rate than that in other countries [9]. It is still unclear why there are so many non-obese type 2 diabetes patients in Japan, and the scientific reasons are under research. 

Animal models wherein obese type 2 diabetes develops after birth with LBW by a eutrophic (high fat) diet already exist [10]. However, there is no animal model wherein non-obese hyperglycemia develops after birth at LBW. The cause of non-obese type 2 diabetes is thought to be insulin resistance due to fat accumulation in the liver and skeletal muscle as visceral or ectopic fat [11]. In humans, the relationship between the development rate of type 2 diabetes and birth weight shows a U-shape [12], suggesting that future visceral fat accumulation occurs regardless of whether birthweight is high or low. However, the mechanisms of insulin resistance and details of body composition are unclear in non-obese type 2 diabetes that develops after birth at LBW. 

The aims of this study were to develop a mouse model with non-obese hyperglycemia that develops after birth at LBW and to clarify the pathogenetic mechanism of non-obese hyperglycemia in our mouse model.

## 2. Materials and Methods

### 2.1. Study Design, Protocol, and Animal Model

This study was carried out in accordance with the ARRIVE guidelines, and the protocols were approved by the Nihon University Institutional Animal Care and Use Committee (protocol nos. AP18MED033-1 [5 July 2019] and AP20MED003-1 [3 April 2020]). ICR mice strains at 12 days of gestation were obtained from Sankyo Labo Service Corporation Inc., Tokyo, Japan. All mice were fed a normal solid diet (moisture: 7.9%, crude fat: 5.1%, crude protein: 23.1%, crude ash: 5.8%, crude fiber: 2.8%, and soluble solids: 55.3% (Oriental Yeast Co., Ltd., Tokyo, Japan) and had access to water ad libitum.

**Figure 1:**
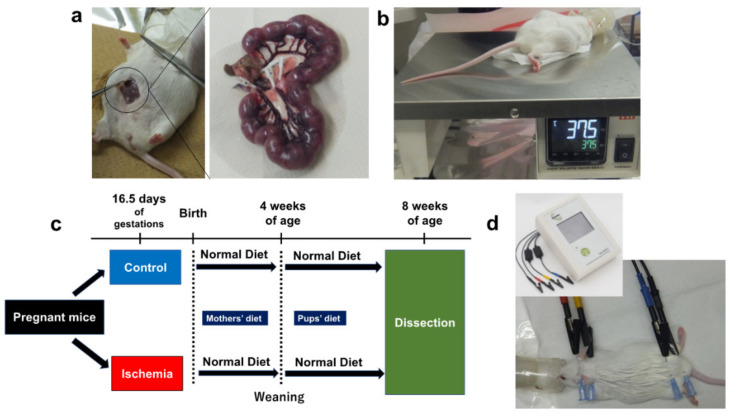
**Experimental procedures**. (**a**) Uterine artery ligation in pregnant mice (ischemia for 15 min). (**b**) Body temperature control on a hot plate (37.5 °C). (**c**) Study flow of this study. (**d**) Body composition measurements using ImpediVET^TM^ (Bioresearch center, Co., Ltd., Nagoya, Japan).

The lower abdomen was incised under isoflurane inhalation anesthesia (induction 5%, maintenance 2%) at 16.5 days of gestation. In the intrauterine ischemia (group I), maternal mice were pre-warmed at 37.5 °C on a hot plate, the uterine artery was exposed and blood flow to the artery was blocked by a clip for 15 min to lead to fetus hypoxia and undernutrition (Figure 1a,b) [13]. The uterine artery was then unclipped, the were fetuses returned into the abdomen of the mother mice, and the abdomen was sutured. The controls (group C) only underwent a lower abdominal incision under similar anesthesia (sham control). Newborn pups were reared under the care of their mothers; female pups from the two groups were weaned at 4 weeks of age after birth and reared on a normal diet until 8 weeks of age (Figure 1c). 

At birth and thereafter, the pups were weighed twice a week until 8 weeks of age. The body weight gain plateaued at approximately 8 weeks of age. Eight-week-old mice represent human adulthood [14]. At 8 weeks of age, body composition was measured, blood was drawn from the heart, and the liver was removed after 12 h of fasting (Figure 1c,d). Fasting blood glucose levels, serum immunoreactive insulin (IRI), body composition, and serum lipoprotein levels were measured at 8 weeks of age. Metabolite analyses were performed on liver tissues at 8 weeks of age. Results were compared between group I and C (*n* = 7 for each group).

### 2.2. Glucose Metabolism Markers

Blood glucose levels after 12 h of fasting were measured using a Stat Strip XP2 (Nipro Corp., Osaka, Japan). Blood was then centrifuged at 3000 rpm for 5 min at room temperature and the serum was stored at −20 °C. Serum IRI levels were measured using a mouse/rat total insulin (high sensitivity) assay kit (Immuno-Biological Laboratories Co., Ltd., Fujioka, Gunma, Japan). Homeostasis model assessment of insulin resistance (HOMA-R) was calculated using the human formula:

HOMA-R = fasting blood glucose (mg/dL) × IRI (µIU/mL)/405

since there is no formula for mice [15].

### 2.3. Body Composition Analyses

Body composition was measured using the bioelectrical impedance analysis method using a body composition analyzer for laboratory animals (ImpediVET^TM^, Bioresearch center, Co., Ltd., Nagoya, Japan) (Figure 1d) [15]. Bioelectrical impedance analysis is used to estimate body composition (such as body fat percentage) by measuring the electrical resistance (bioimpedance) of biological tissues. Adipose tissue conducts almost no electricity, while muscle and other tissues that contain many electrolytes easily conduct electricity. The ratio of fat to other tissues can be estimated by measuring electrical resistance [16]. Fat mass percentage and fat-free mass percentage were measured. Fat mass (g) (1) or fat-free mass (g) (2) were calculated using the following formula:
(1)Fat mass (g) = eight-week-old body weight (g) × fat mass percentage/100(2)Fat-free mass (g) = eight-week-old body weight (g) × fat-free mass percentage/100

### 2.4. Serum Lipoprotein Levels

Cholesterol and triglyceride profiles in serum lipoproteins were analyzed using a previously described gel-permeation high-performance liquid chromatography method (LipoSEARCH^®^; Skylight Biotech, Akita, Japan) [17,18,19]. Cholesterol and triglyceride levels of total- and major classes of lipoproteins (high density lipoprotein, HDL; low density lipoprotein, LDL; very low-density lipoprotein, VLDL) were defined using component peak analyses based on lipoprotein particle sizes using the Gaussian curve fitting technique [18].

### 2.5. Metabolite Analyses in Liver

Metabolites were extracted using the following methods: approximately 50 mg of frozen liver tissue from female mice (8 weeks of age, *n* = 3 each group) was placed in a homogenization tube along with zirconia beads (5 mm φ and 3 mm φ). Next, 1500 µL of 50% acetonitrile/Milli-Q water containing internal standards (H3304-1002, Human Metabolome Technologies, Inc. (HMT), Tsuruoka, Yamagata, Japan) was added, followed by two cycles of tissue homogenization using a bead shaker at 1500 rpm for 120 s at 4 °C each (Shake Master NEO, Bio Medical Science, Tokyo, Japan). The homogenate was centrifuged at 2300× *g* for 5 min at 4 °C. The upper aqueous layer (400 µL) was centrifugally filtered at 9100× *g* for 120 min at 4 °C using a Millipore 5-kDa cutoff filter (Human Metabolome Technologies, Inc.) to remove macromolecules. Under vacuum, the filtrate was evaporated to dryness and redissolved in 50 µL of Milli-Q water for the metabolome analysis. 

Metabolome analyses were conducted using capillary electrophoresis time-of-flight mass spectrometry, as previously described [20,21]. Briefly, capillary electrophoresis time-of-flight mass spectrometry analysis was performed using an Agilent CE capillary electrophoresis system (Agilent Technologies, Inc., Santa Clara, CA, USA). The spectrometer was scanned at 50–1000 *m*/*z* and peaks were extracted by integration software (Keio University, Tsuruoka, Yamagata, Japan) to obtain the following data; *m*/*z*, migration time, and peak area [22]. The peaks were determined according to the metabolite database based on their *m*/*z* values and migration times. Peak areas were normalized using internal standards and sample volume, then relative levels of the metabolites were obtained.

Principal component analysis and hierarchical cluster analysis were performed, as previously described [23]. Detected metabolites were plotted on metabolic pathway maps, as previously described [24].

### 2.6. Statistical Analyses

Data are expressed as the mean ± standard error of the mean. Comparisons between the two groups were performed with the Mann-Whitney U test or Welch’s *t* test as appropriate using JMP ver. 14 (SAS Institute, Cary, NC, USA). A *p* value < 0.05 was considered a significant difference.

## 3. Results

### 3.1. Birth Weight and Changes in Body Weight Gain

**Figure 2:**
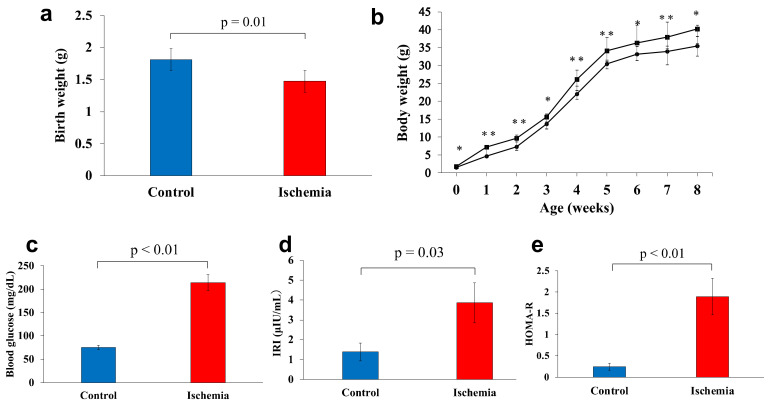
**Body weight and glucose metabolism markers**. (**a**) Birthweight was measured on the first day after birth. (**b**) Changes in weight gain from birth to 8 weeks of age (●: Ischemia, ■: Control). (**c**) Fasting blood glucose levels. (**d**) Serum immunoreactive insulin levels. (**e**) Homeostasis model assessment of insulin resistance levels. Data are shown as the mean ± standard error of the mean (*n* = 7 per group). * *p* < 0.05, ** *p* < 0.01.

Birthweight was significantly lower in group I (1.5 g) than that in group C (1.8 g) (*p* = 0.01) (Figure 2a). The mean body weights of groups I and C at 1, 2, 3, 4, 5, 6, 7, and 8 weeks of age were: 4.7 and 7.2 g, *p* < 0.01; 7.3 and 9.7 g, *p* < 0.01; 14.0 and 15.5 g, *p* = 0.03; 22.1 and 26.1 g, *p* < 0.01; 30.5 and 34.1 g, *p* < 0.01; 33.2 and 36.3 g, *p* = 0.02; 33.9 and 37.9 g, *p* < 0.05; and 35.5 and 40.2 g, *p* = 0.01, respectively. Group I had a LBW and was consistently underweight thereafter, even at 8 weeks of age (Figure 2b).

### 3.2. Glucose Metabolism Markers

The mean fasting blood glucose level at 8 weeks of age was significantly higher in group I compared with group C (196.9 and 75.0 mg/dL, respectively) (*p* < 0.01). The mean levels of IRI and HOMA-R were significantly higher in group I (3.9 µIU/mL and 1.9, respectively) compared with group C (1.4 µIU/mL and 0.3, respectively) (*p* = 0.03, *p* < 0.01, respectively; Figure 2c–e).

### 3.3. Body Composition

**Figure 3:**
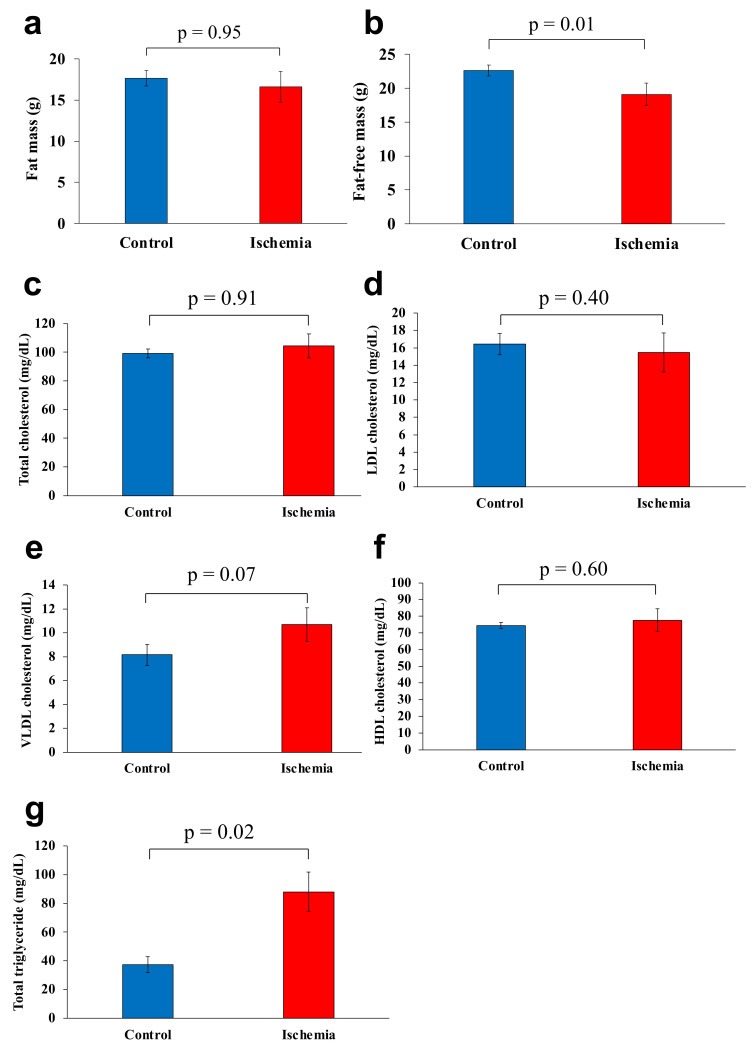
**Body composition and serum lipoprotein levels**. (**a**) Fat mass. (**b**) Fat-free mass. (**c**) Total cholesterol. (**d**) LDL cholesterol. (**e**) VLDL cholesterol. (**f**) HDL cholesterol. (**g**) Total triglyceride. Data are shown as the mean ± standard error of the mean (*n* = 7 per group). HDL, high-density lipoprotein; LDL, low-density lipoprotein; VLDL, very-low-density lipoprotein.

There was no significant difference between the mean fat mass of group I and group C (16.6 and 17.7 g, respectively) (*p* = 0.95, Figure 3a). Meanwhile, the mean fat-free mass was significantly lower in group I than that of group C (19.1 and 22.6 g, respectively) (*p* = 0.01, Figure 3b).

### 3.4. Serum Lipoprotein Levels

Mean total cholesterol, LDL cholesterol, VLDL cholesterol, and HDL cholesterol levels were 104.4 mg/dL, 15.5 mg/dL, 10.7 mg/dL, and 77.6 mg/dL, respectively in group I and 99.3 mg/dL, 16.5 mg/dL, 8.2 mg/dL, and 74.3 mg/dL, respectively in group C, with no significant differences between the two groups (Figure 3c–f). Total triglyceride level was significantly higher in Group I (88.1 mg/dL) than that of group C (37.3 mg/dL) (*p* < 0.05; Figure 3g). 

### 3.5. Liver Metabolite Analyses

**Figure 4:**
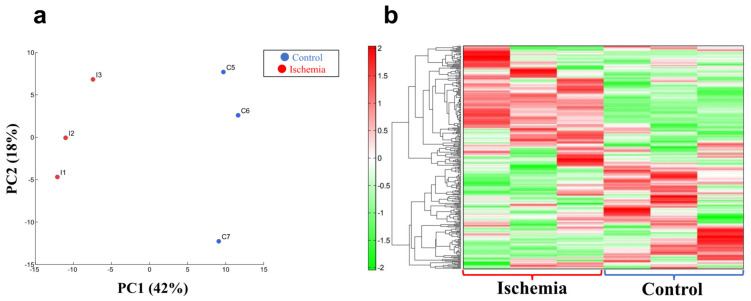
**Metabolite analyses in liver tissue**. (**a**) Principal component (PC) analysis. (**b**) Heat map display of the hierarchical cluster analysis. *n* = 3 per group.

A clear difference was found between group I and group C in the principal component analysis and the heat map display of the hierarchical cluster analysis (*n* = 3 for each group, Figure 4a,b; Supplementary Tables S1 and S2).

**Figure 5:**
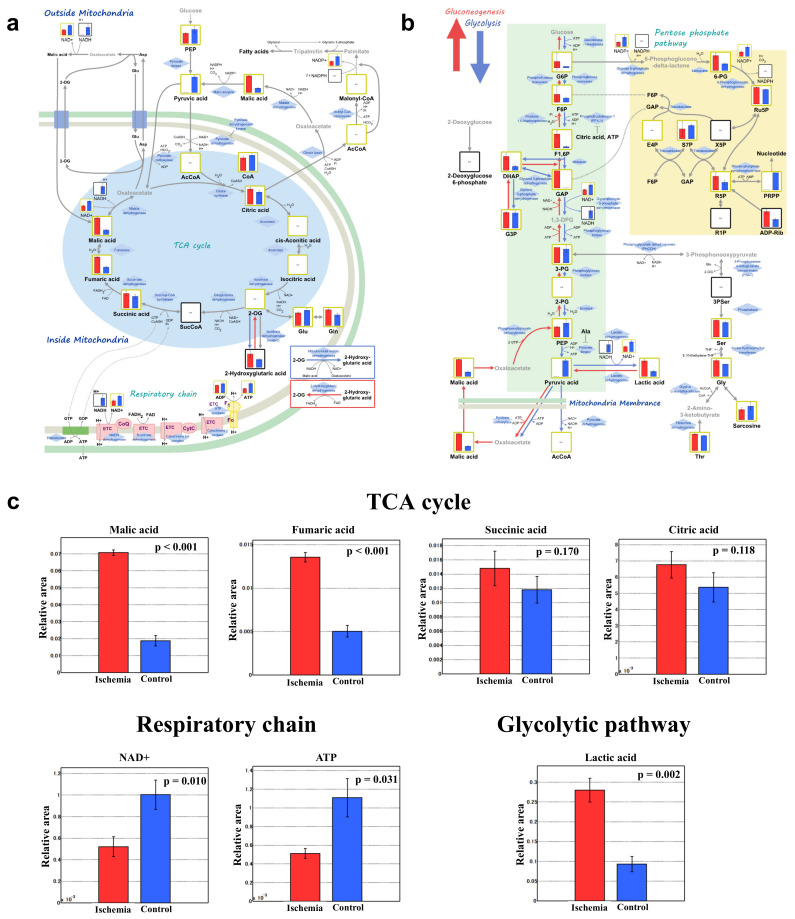
**Comprehensive comparative analysis between the ischemia and control groups for the TCA cycle, respiratory chain, and glycolytic pathway**. (**a**) TCA cycle and respiratory chain. (**b**) Glycolytic pathway. (**c**) Important metabolites. Red and blue bars show group I and C, respectively. ATP, adenosine triphosphate; NAD^+^; nicotinamide adenine dinucleotide; TCA, tricarboxylic acid; *n* = 3 per group.

Comparative analysis of the tricarboxylic acid (TCA) cycle, respiratory chain, and glycolytic pathway between group I and C are shown in Figure 5a,b, and Supplementary Table S3. Malic acid, fumaric acid, succinic acid, and citric acid were higher in group I than group C (*p* < 0.001, *p* < 0.001, *p* = 0.170, and *p* = 0.118, respectively; Figure 5c). Respiratory chain analyses showed that nicotinamide adenine dinucleotide (NAD^+^) and adenosine triphosphate (ATP) were significantly lower in group I than that of group C (*p* = 0.010 and *p* = 0.031, respectively). Meanwhile, lactic acid in the glycolytic pathway was significantly higher in group I than that in group C (*p* = 0.002). Representative oxidative stress markers 3-indoxylsulfuric acid, cysteine, and S-adenosylmethionine were significantly higher in group I than that in group C (*p* < 0.001, *p* < 0.05, and *p* < 0.01, respectively; Table 1).

## 4. Discussion

**Figure 6:**
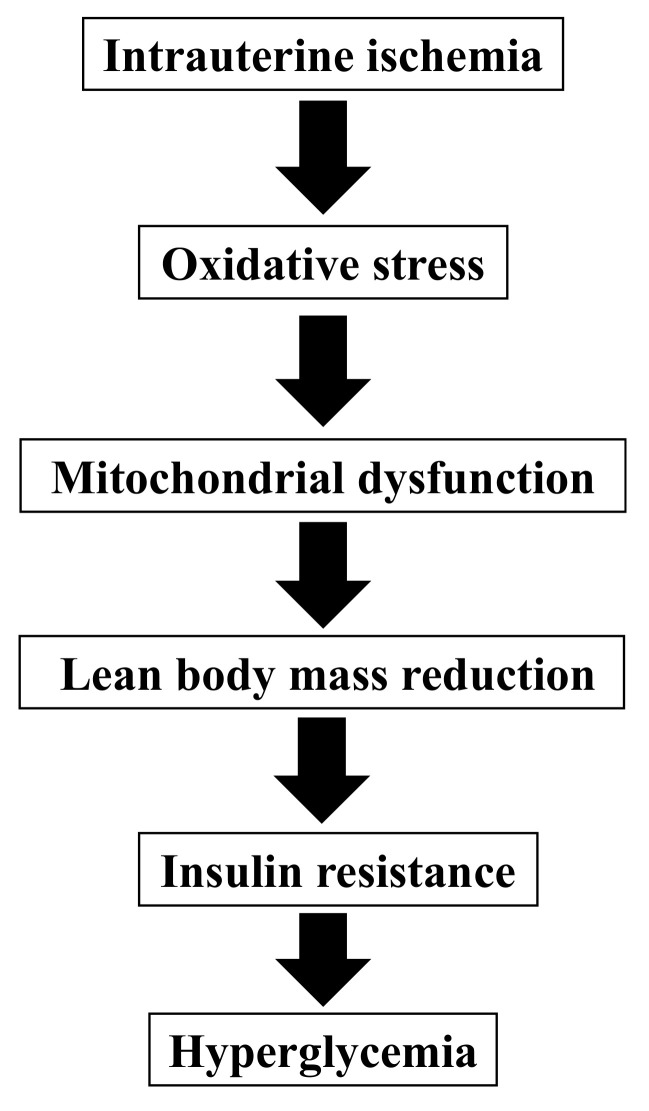
A theory for the pathogenesis of non-obese hyperglycemia after birth with low birthweight in our model.

In clinical practice, patients born with LBW can develop type 2 diabetes without significant postnatal obesity; however, animal models have not yet been developed. In this study, we confirmed that our mouse model using intrauterine ischemia by transiently blocking blood flow of uterine arteries in pregnant mice yields non-obese hyperglycemia in young adulthood after birth with LBW. Total cholesterol, LDL cholesterol, VLDL cholesterol, and HDL cholesterol levels were not significantly different from the controls. Reduced lean body mass and mitochondrial dysfunction contributed to the increased myogenic insulin resistance of non-obese hyperglycemia (Figure 6).

### 4.1. Mice Model Born with LBW

Intrauterine malnutrition, such as ligation of bilateral uterine arteries in pregnant rats or food restriction of pregnant animals, can cause fetal growth restriction [25,26,27,28]. There are animal models that develop hyperglycemia with adulthood obesity [10] and that remain underweight in adulthood but do not develop hyperglycemia [29]. However, this is the first animal model that develops hyperglycemia after birth with LBW without developing adulthood obesity on a normal diet. Further studies are needed to investigate if the pathogenesis of our mouse model is related to that of human non-obese type 2 diabetic patients.

### 4.2. Myogenic Insulin Resistance

Group I had significantly lower fat-free mass than that of group C, although there was no difference in fat mass between the groups. This may be due to decreased muscle mass in group I since this group was not obese. Patients born with LBW tend to have low muscle mass in adulthood [30] and their basal metabolism is low [31] which leads to visceral fat accumulation, decreased adiponectin secretion, and insulin resistance [32]. Our animal model showed myogenic insulin resistance due to reduced muscle mass which is considered one of the causes of non-obese diabetes.

### 4.3. Mitochondrial Dysfunction

**Figure 7:**
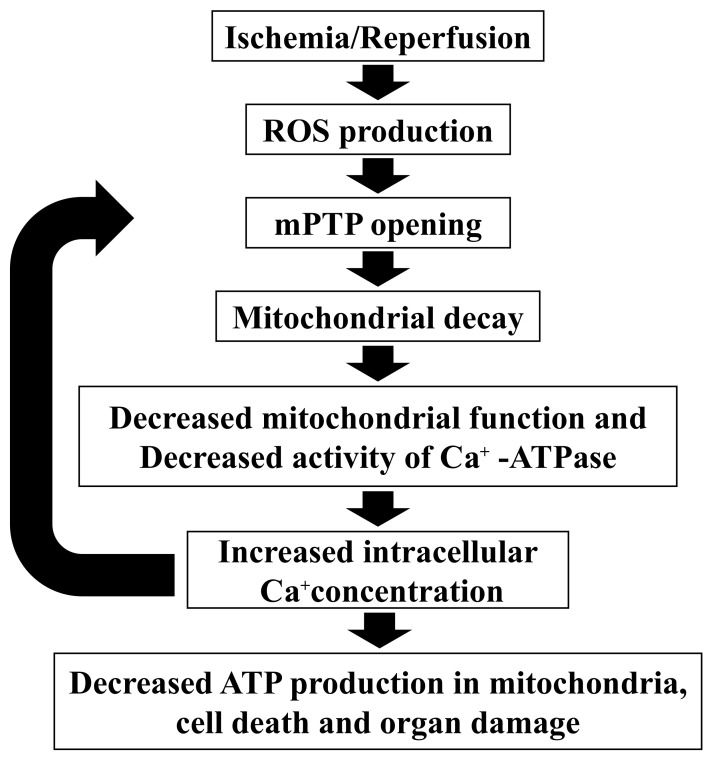
**Mitochondria dysfunction by ischemia and reperfusion**. ATP, adenosine triphosphate; mPTP, mitochondrial permeability transition pore; ROS, reactive oxygen species.

Mitochondria are the site of energy production such as ATP; therefore, mitochondrial dysfunction decreases ATP production. Lactic acid increases since ATP is produced through anaerobic glycolysis [33]. NAD^+^ is one of the cofactors for energy production in mitochondria and is also reduced by mitochondrial dysfunction [34]. In this study, group I liver metabolite analyses showed decreased mitochondrial function through decreased ATP production, increased lactic acid, and decreased NAD^+^. In addition, 3-indoxylsulfuric acid (an oxidative stress molecule) was significantly higher in group I compared with group C. Ischemia and reperfusion produce oxidative stress, such as reactive oxygen species, resulting in decreased mitochondrial function [35,36,37,38]; this suggests that the intrauterine ischemia in the present model caused mitochondrial dysfunction by the same mechanism (Figure 7). 

Diabetes development is strongly associated with mitochondrial dysfunction in skeletal muscle and liver; therefore, mitochondrial dysfunction can cause insulin resistance [39]. The clinical features of mitochondrial diabetes include short stature and non-obesity [40]. The development of mitochondrial diabetes is at a relatively young age (in the 30s), and the maternal inheritance of diabetes mellitus is 59%; therefore, not all cases are maternally inherited. Mitochondrial diabetes varies from insulin deficiency to insulin resistance. It is thought that autoimmune mechanisms are less likely to be involved. Other reports demonstrated a link between insulin resistance and mitochondrial dysfunction in the elderly [41] and mitochondrial dysfunction in a close relative of a diabetic patient [41]. Furthermore, type 2 diabetic patients have decreased expression of mitochondrial respiratory chain complexes or mitochondrial metabolism-related genes compared with healthy controls [42,43] while continuous physical activity improves insulin resistance and mitochondrial dysfunction in type 2 diabetic patients and obese individuals [44]. In addition, decreased mitochondrial DNA in peripheral blood cells correlates with insulin resistance [45] suggesting that quantitative or qualitative mitochondrial decline may be involved in the development of non-obese type 2 diabetes. Mitochondrial dysfunction causes muscle atrophy [46] and may be associated with insulin resistance due to reduced muscle mass which is the cause of non-obese type 2 diabetes.

### 4.4. Other Pathogeneses

As other pathogeneses, increased insulin clearance [47], decreased pancreatic β-cell function [48], and enlarged fat cells [49] have been reported in the cause of non-obese type 2 diabetes. It is necessary to study if these causes exist in this model using biochemical, genetic, and histopathological analyses.

### 4.5. Limitations

There were several limitations in this study. First, there was a small number of animals due to ethical issues. Second, only female mice were included because many male mice died due to the intrauterine ischemia. The results of male mice showed a similar trend; however, the relationship was not as significant as those of females. Fewer male mice used in the experiments may have contributed to a less significant difference. Mitochondria are maternally inherited and may be more likely to appear as a female phenotype; however, further studies are needed using large sample sizes in both sexes. Third, visceral fat accumulation was not assessed by any imaging. Fourth, intrauterine ischemia is a cause of LBW at birth, but not of all LBW causes. Finally, since the equation of HOMA-R generally use is for humans, it is necessary to verify whether the results of this formula really represent insulin resistance in mice.

## 5. Conclusions

This mouse model showed non-obese hyperglycemia in young adulthood after birth with LBW due to transient intrauterine ischemia. A pathogenetic mechanism may involve increased myogenic insulin resistance by mitochondrial dysfunction. In the future, using this model, preventive and therapeutic strategies for non-obese hyperglycemia will be studied, such as the use of growth hormone, whey protein, or Chinese medicine, and non-invasive insulin therapy [50].

## 6. Patents

A method for producing a mouse model that develops non-obese type 2 diabetic in young adulthood after birth with LBW due to transient intrauterine ischemia was lodged with the Japanese Patent Office on 6 July 2020, by Nobuhiko Nagano, Ichiro Morioka, Shoichi Shimizu, and Daichi Katayama (application number: 2020-116354).

## Figures and Tables

**Table 1:** Oxidative stress markers.

		Comparative Analysis	
		Group I vs. C	
Compound Name	Compound Name	Ratio ^†^	*p*-Value ^‖^
Oxidative stress	3-indoxylsulfuric acid	2.0	<0.001
	Cys	3.0	0.011
	*S*-adenosylmethionine Ergothioneine *N*,*N*-dimethylglycine	1.7 0.7 0.9	0.003 0.061 0.683

^†^ The ratio of the detected mean values between the two groups. ^‖^ Welch’s *t*-test.

## Data Availability

The data that support the findings of this study are available from the corresponding author upon reasonable request.

## Supplementary Materials

The following supporting information can be downloaded at: https://www.mdpi.com/article/10.3390/biomedicines10071642/s1, Table S1: principal component score; Table S2: metabolites and principal component score; Table S3: comparative analysis.

## References

[1] Barker D.J., Osmond C. (1986). Infant mortality, childhood nutrition, and ischaemic heart disease in England and Wales. Lancet.

[2] Gluckman P.D., Hanson M.A. (2004). Living with the past: Evolution, development, and patterns of disease. Science.

[3] De Boo H.A., Harding J.E. (2006). The developmental origins of adult disease (Barker) hypothesis. Aust. N. Z. J. Obstet. Gynaecol..

[4] Ministry of Health, Labor and Welfare in Japan Vital Statistics in Japan in 2017. https://www.mhlw.go.jp/toukei/list/81-1.html.

[5] The World Bank Low-Birthweight Babies (% of Birth). https://data.worldbank.org/indicator/SH.STA.BRTW.ZS.

[6] Kuwabara R., Urakami T., Yoshida K., Morioka I. (2020). Case of type 2 diabetes possibly caused by excessive accumulation of visceral fat in a child born small-for-gestational age. J. Diabetes Investig..

[7] Sone H., Ito H., Ohashi Y., Akanuma Y., Yamada N., Japan Diabetes Complication Study Group (2003). Obesity and type 2 diabetes in Japanese patients. Lancet.

[8] The Examination Committee of Criteria for ‘Obesity Disease’ in Japan, Japan Society for the Study of Obesity (2002). New criteria for ‘obesity disease’ in Japan. Circ. J..

[9] Urakami T., Morimoto S., Nitadori Y., Harada K., Owada M., Kitagawa T. (2007). Urine glucose screening program at schools in Japan to detect children with diabetes and its outcome-incidence and clinical characteristics of childhood type 2 diabetes in Japan. Pediatr. Res..

[10] Simmons R.A., Templeton L.J., Gertz S.J. (2001). Intrauterine growth retardation leads to the development of type 2 diabetes in the rat. Diabetes.

[11] Takeno K., Tamura Y., Kawaguchi M., Kakehi S., Watanabe T., Funayama T., Furukawa Y., Kaga H., Yamamoto R., Kim M. (2016). Relation between insulin sensitivity and metabolic abnormalities in Japanese men with BMI of 23–25 kg/m^2^. J. Clin. Endocrinol. Metab..

[12] Spalding K.L., Arner E., Westermark P.O., Bernard S., Buchholz B.A., Bergmann O., Blomqvist L., Hoffstedt J., Näslund E., Britton T. (2008). Dynamics of fat cell turnover in humans. Nature.

[13] Kubo K.-I., Deguchi K., Nagai T., Ito Y., Yoshida K., Endo T., Benner S., Shan W., Kitazawa A., Aramaki M. (2017). Association of impaired neuronal migration with cognitive deficits in extremely preterm infants. JCI Insight.

[14] Kimura K., Takeuchi K. (1986). Growth of the Jcl: ICR mouse. Okajimas Folia Anat. Jpn..

[15] Matthews D.R., Hosker J.P., Rudenski A.S., Naylor B.A., Treacher D.F., Turner R.C. (1985). Homeostasis model assessment: Insulin resistance and beta-cell function from fasting plasma glucose and insulin concentrations in man. Diabetologia.

[16] Lukaski H.C., Johnson P.E., Bolonchuk W.W., Lykken G.I. (1985). Assessment of fat free mass using bioelectrical impedance measurements of the human body. Am. J. Clin. Nutr..

[17] Okazaki M., Yamashita S. (2016). Recent advances in analytical methods on lipoprotein subclasses: Calculation of particle numbers from lipid levels by gel permeation HPLC using “Spherical Particle Model”. J. Oleo Sci..

[18] Usui S., Hara Y., Hosaki S., Okazaki M. (2002). A new on-line dual enzymatic method for simultaneous quantification of cholesterol and triglycerides in lipoproteins by HPLC. J. Lipid Res..

[19] Okazaki M., Usui S., Ishigami M., Sakai N., Nakamura T., Matsuzawa Y., Yamashita S. (2005). Identification of unique lipoprotein subclasses for visceral obesity by component analysis of cholesterol profile in high-performance liquid chromatography. Arterioscler. Thromb. Vasc. Biol..

[20] Ohashi Y., Hirayama A., Ishikawa T., Nakamura S., Shimizu K., Ueno Y., Tomita M., Soga T. (2008). Depiction of metabolome changes in histidine-starved *Escherichia coli* by CE-TOFMS. Mol. Biosyst..

[21] Ooga T., Sato H., Nagashima A., Sasaki K., Tomita M., Soga T., Ohashi Y. (2011). Metabolomic anatomy of an animal model revealing homeostatic imbalances in dyslipidaemia. Mol. Biosyst..

[22] Sugimoto M., Wong D.T., Hirayama A., Soga T., Tomita M. (2010). Capillary electrophoresis mass spectrometry-based saliva metabolomics identified oral, breast and pancreatic cancer-specific profiles. Metabolomics.

[23] Yamamoto H., Fujimori T., Sato H., Ishikawa G., Kami K., Ohashi Y. (2014). Statistical hypothesis testing of factor loading in principal component analysis and its application to metabolite set enrichment analysis. BMC Bioinform..

[24] Junker B.H., Klukas C., Schreiber F. (2006). VANTED: A system for advanced data analysis and visualization in the context of biological networks. BMC Bioinform..

[25] Wigglesworth J.S. (1964). Experimental growth retardation in the foetal rat. J. Pathol. Bacteriol..

[26] Garofano A., Czernichow P., Breant B. (1997). In utero undernutrition impairs rat beta-cell development. Diabetologia.

[27] Ozaki T., Nishina H., Hanson M.A., Poston L. (2001). Dietary restriction in pregnant rats causes gender-related hypertension and vascular dysfunction in offspring. J. Physiol..

[28] Jimenez-Chillaron J.C., Hernandez-Valencia M., Reamer C., Fisher S., Joszi A., Hirshman M., Oge A., Walrond S., Przybyla R., Boozer C. (2005). β-cell secretory dysfunction in the pathogenesis of low birth weight-associated diabetes: A murine model. Diabetes.

[29] Ogata E.S., Bussey M.E., Finley S. (1986). Altered gas exchange, limited glucose and branched chain amino acids, and hypoinsulinism retard fetal growth in the rat. Metabolism.

[30] Ylihärsilä H., Kajantie E., Osmond C., Forsén T., Barker D.J., Eriksson J.G. (2007). Birth size, adult body composition and muscle strength in later life. Int. J. Obes..

[31] Matinolli H.M., Hovi P., Männistö S., Sipola-Leppänen M., Eriksson J.G., Mäkitie O., Järvenpää A.L., Andersson S., Kajantie E. (2015). Early protein intake is associated with body composition and resting energy expenditure in young adults born with very low birth weight. J. Nutr..

[32] Cho W.K., Suh B.K. (2016). Catch-up growth and catch-up fat in children born small for gestational age. Korean J. Pediatr..

[33] Feng Z., Hanson R.W., Berger N.A., Trubitsyn A. (2016). Reprogramming of energy metabolism as a driver of aging. Oncotarget.

[34] Imai S.-I., Guarente L. (2014). NAD^+^ and sirtuins in aging and disease. Trends Cell Biol..

[35] Granger D.N., Kvietys P.R. (2015). Reperfusion injury and reactive oxygen species: The evolution of a concept. Redox Biol..

[36] Crabtree M.J., Hale A.B., Channon K.M. (2011). Dihydrofolate reductase protects endothelial nitric oxide synthase from uncoupling in tetrahydrobiopterin deficiency. Free Radic. Biol. Med..

[37] Rasola A., Bernardi P. (2007). The mitochondrial permeability transition pore and its involvement in cell death and in disease pathogenesis. Apoptosis.

[38] Yu N., Wang S., Wang P., Li Y., Li S., Chen H., Wang T. (2012). The calcium uniporter regulates the permeability transition pore in isolated cortical mitochondria. Neural Regen. Res..

[39] Sangwung P., Petersen K.F., Shulman G.I., Knowles J.W. (2020). Mitochondrial dysfunction, insulin resistance, and potential genetic implications. Endocrinology.

[40] Zhao Y., Zhang Y., Qi M., Ping F., Zhang H., Xu L., Li W., Li Y. (2022). The role of lactate exercise test and fasting plasma c-peptide levels in the diagnosis of mitochondrial diabetes: Analysis of clinical characteristics of 12 patients with mitochondrial diabetes in a single center with long-term follow-up. Front. Endocrinol..

[41] Petersen K.F., Befroy D., Dufour S., Dziura J., Ariyan C., Rothmann D.L., DiPietro L., Cline G.W., Shulman G.I. (2003). Mitochondrial dysfunction in the elderly: Possible role in insulin resistance. Science.

[42] Petersen K.F., Dufour S., Befroy D., Garcia R., Shulman G.I. (2004). Impaired mitochondrial activity in the insulin-resistant offspring of patients with type 2 diabetes. N. Engl. J. Med..

[43] Kelley D.E., He J., Menshikova E.V., Ritov V.B. (2002). Dysfunction of mitochondria in human skeletal muscle in type 2 diabetes. Diabetes.

[44] Toledo F.G., Menshikova E.V., Ritov V.B., Azuma K., Radikova Z., DeLany J., Kelley D.E. (2007). Effects of physical activity and weight loss on skeletal muscle mitochondria and relationship with glucose control in type 2 diabetes. Diabetes.

[45] Song J., Oh J.Y., Sung Y.A., Pak Y., Park K.S., Lee H.K. (2001). Peripheral blood mitochondrial DNA content is related to insulin sensitivity in offspring of type 2 diabetic patients. Diabetes Care.

[46] Wang X., Li H., Zheng A., Yang L., Liu J., Chen C., Tang Y., Zou X., Li Y., Long J. (2014). Mitochondrial dysfunction-associated OPA1 cleavage contributes to muscle degeneration: Preventative effect of hydroxytyrosol acetate. Cell Death Dis..

[47] Sugiyama S., Jinnouchi H., Hieshima K., Kurinami K., Jinnouchi K. (2021). A non-obese, treatment-naive Japanese diabetic patient with elevated insulin clearance and hyperglycemia under enhanced insulin sensitivity and increased insulin secretion: Elevated insulin clearance as type 2 Japanese diabetes mellitus (T2JDM). Cureus.

[48] Furuta M., Tamai M., Hanabusa T., Yamamoto Y., Nanjo K., Sanke T. (2006). Serum adiponectin is associated with fasting serum C-peptide in non-obese diabetic patients. Diabetes Res. Clin. Pract..

[49] Rattarasarn C. (2018). Dysregulated lipid storage and its relationship with insulin resistance and cardiovascular risk factors in non-obese Asian patients with type 2 diabetes. Adipocyte.

[50] Sabbagh F., Muhamad I.I., Niazmand R., Dikshit P.K., Kim B.S. (2022). Recent progress in polymeric non-invasive insulin delivery. Int. J. Biol. Macromol..

