# Supplementary material for: A Non-Obese Hyperglycemic Mouse Model that Develops after Birth with Low Birthweight
Source: Biomedicines. 2022 Jul 8;10(7):1642. doi: 10.3390/biomedicines10071642 (PMC9312481; doi:10.3390/biomedicines10071642)
Supplement: Supplementary file 1 [file biomedicines-10-01642-s001.zip › Supplementary Table 1.pdf]

Supplementary Table S1. Principal component score

	Contribution rate (%)	Group I			Group C		
		I1	I2	I3	C1	C2	C3
PC1	41.99	-12.09	-11.01	-7.41	9.73	11.66	9.12
PC2	18.80	-4.70	-0.08	6.81	7.68	2.58	-12.28
PC3	15.49	8.01	0.62	-10.21	2.82	4.72	-5.97
PC4	13.40	-6.54	10.96	-5.23	2.57	-2.39	0.63
PC5	10.32	-2.56	2.77	0.94	-8.02	8.60	-1.73

PC, principal component
