# Supplementary material for: A Non-Obese Hyperglycemic Mouse Model that Develops after Birth with Low Birthweight
Source: Biomedicines. 2022 Jul 8;10(7):1642. doi: 10.3390/biomedicines10071642 (PMC9312481; doi:10.3390/biomedicines10071642)
Supplement: Supplementary file 1 [file biomedicines-10-01642-s001.zip › Supplementary Table 2.pdf]

Supplementary Table S2. Metabolites and principal component score

	Compound name	PubChem CID	HMDB ID	*m/z*	MT/RT	PC1	PC2
A_0003	Pyruvic acid	1060	HMDB0000243	87.009	10.49	9.9E-01	-2.2E-02
A_0004	Butyric acid<br>Isobutyric acid	264<br>6590	HMDB0000039<br>HMDB0001873	87.045	8.31	-3.2E-01	4.4E-01
A_0005	Lactic acid	612	HMDB0000190,HMDB0001311	89.024	9.00	-9.5E-01	1.3E-01
A_0006	3-Hydroxybutyric acid	441	HMDB0000011,HMDB0000357,HMDB0000442	103.039	8.04	8.6E-01	3.4E-01
A_0007	2-Hydroxybutyric acid	440864	HMDB0000008	103.040	8.24	7.4E-01	5.3E-01
A_0008	Fumaric acid	444972	HMDB0000134	115.003	17.57	-9.8E-01	3.3E-02
A_0010	Succinic acid	1110	HMDB0000254	117.019	15.52	-5.5E-01	4.3E-01
A_0011	β-Hydroxyisovaleric acid	69362	HMDB0000754	117.056	7.63	2.3E-01	1.3E-01
A_0012	XA0003			124.991	10.24	-5.3E-01	-7.9E-01
A_0013	Isethionic acid	7866	HMDB0003903	124.991	9.55	8.1E-01	2.2E-01
A_0014	5-Oxoproline	7405	HMDB0000267	128.035	7.97	9.1E-01	-1.1E-01
A_0015	*N*-Acetylalanine	88064	HMDB0000766	130.051	7.50	-7.9E-01	4.2E-01
A_0016	Malic acid	525	HMDB0000156,HMDB0000744	133.013	15.73	-9.9E-01	3.5E-02
A_0017	Threonic acid	5460407	HMDB0000943	135.030	7.79	4.7E-01	4.2E-01
A_0018	Ethanolamine phosphate	1015	HMDB0000224	140.011	6.79	8.9E-01	4.0E-01
A_0019	Octanoic acid	379	HMDB0000482	143.107	7.11	4.2E-01	5.0E-01
A_0020	4-Acetamidobutanoic acid	18189	HMDB0003681	144.066	7.29	5.1E-01	1.7E-01
A_0022	2-Hydroxyglutaric acid	43	HMDB0000606,HMDB0000694	147.029	13.32	-8.6E-01	-2.6E-01
A_0023	Cysteinesulfinic acid	1549098	HMDB0000996	152.000	8.01	9.0E-01	-2.9E-01
A_0024	Pelargonic acid	8158	HMDB0000847	157.123	6.93	2.6E-01	5.3E-01
A_0025	Terephthalic acid	7489	HMDB0002428	165.018	13.08	-4.1E-01	-2.7E-01
A_0026	XA0012			166.018	7.89	4.2E-01	-4.0E-01
A_0027	Phosphoenolpyruvic acid	1005	HMDB0000263	166.974	15.58	6.7E-01	-5.9E-01
A_0028	Uric acid	1175	HMDB0000289	167.021	7.50	6.8E-01	6.7E-01
A_0029	Dihydroxyacetone phosphate	668	HMDB0001473	168.990	10.43	-9.6E-01	-7.7E-02
A_0030	Glyceraldehyde 3-phosphate	729	HMDB0001112	168.991	9.66	-5.3E-01	-3.0E-01
A_0031	Glycerol 3-phosphate	439162	HMDB0000126	171.006	10.00	-6.8E-02	3.3E-01
A_0032	Decanoic acid	2969	HMDB0000511	171.140	6.77	2.9E-01	-1.2E-01
A_0033	Isovalerylalanine<br>*N*-Acetylleucine	129285<br>70912	HMDB0000747<br>HMDB0011756	172.097	6.80	-3.8E-01	-6.1E-02
A_0035	*N*-Acetylasparagine	99715	HMDB0006028	173.057	7.11	1.6E-01	5.2E-01
A_0036	Suberic acid	10457	HMDB0000893	173.084	10.57	-3.0E-01	3.7E-01
A_0037	*N*-Acetylaspartic acid	65065	HMDB0000812	174.041	11.76	-9.1E-02	6.9E-01
A_0038	Ascorbic acid	54670067	HMDB0000044	175.024	7.18	-3.3E-02	7.0E-01
A_0039	Allantoic acid	203	HMDB0001209	175.046	7.33	-1.3E-01	-4.2E-01
A_0040	3-Phosphoglyceric acid	439183	HMDB0000807	184.984	14.82	7.1E-01	9.6E-02
A_0042	XA0017			186.114	6.68	6.0E-01	-7.0E-03
A_0043	*N*-Acetylglutamine	25561	HMDB0006029	187.073	6.86	-7.9E-01	5.7E-02
A_0044	Azelaic acid	2266	HMDB0000784	187.097	10.07	-2.9E-01	4.3E-01
A_0045	*N*-Acetylglutamic acid	70914	HMDB0001138	188.056	10.82	-3.4E-01	-3.1E-01
A_0046	*N*-Acetylmethionine	448580	HMDB0011745	190.055	6.89	-9.1E-01	1.6E-01
A_0048	XA0019			191.019	7.05	9.1E-01	1.5E-01
A_0049	Citric acid	311	HMDB0000094	191.020	17.85	-7.4E-01	-4.4E-01
A_0050	Quinic acid	6508	HMDB0003072	191.065	6.55	-3.0E-02	-1.4E-01
A_0051	*N*-(*o*-Toluoyl)glycine	91637	HMDB0011723	192.065	6.91	6.1E-01	-7.2E-01
A_0052	Phenaceturic acid	68144	HMDB0000821	192.066	6.97	-4.2E-01	-4.9E-01
A_0053	Galacturonic acid-1<br>Glucuronic acid-1	439215<br>94715	HMDB0002545<br>HMDB0000127	193.035	7.01	-4.7E-01	-5.2E-01
A_0054	Galacturonic acid-2<br>Glucuronic acid-2	439215<br>94715	HMDB0002545<br>HMDB0000127	193.036	6.87	4.6E-01	-2.5E-02
A_0055	Gluconic acid	10690	HMDB0000625	195.050	6.96	8.8E-01	3.0E-01
A_0057	Lauric acid	3893	HMDB0000638	199.171	6.53	7.6E-01	4.5E-01
A_0058	Mucic acid	3037582	HMDB0000639	209.029	11.41	1.4E-01	-2.1E-01
A_0059	3-Indoxylsulfuric acid	10258	HMDB0000682	212.001	8.01	-9.7E-01	1.7E-01
A_0060	Pantothenic acid	6613	HMDB0000210	218.102	6.52	6.0E-01	-1.8E-01
A_0061	Ethyl glucuronide	18392195	HMDB0010325	221.066	6.61	4.7E-01	-8.5E-01
A_0062	Myristoleic acid	5281119	HMDB0002000	225.185	6.38	7.5E-01	6.0E-01
A_0063	Myristic acid	11005	HMDB0000806	227.202	6.36	2.9E-02	7.1E-01
A_0064	Ribulose 5-phosphate	439184	HMDB0000618	229.012	9.14	-9.3E-01	-7.3E-02
A_0065	Ribose 5-phosphate	439167	HMDB0001548	229.012	8.80	-8.2E-01	-7.7E-03
A_0066	XA0033			242.080	6.47	9.5E-02	-2.6E-01
A_0067	Ascorbate 2-sulfate	54676864		254.982	11.59	-7.8E-01	4.0E-01
A_0068	XA0035			254.983	11.08	-9.0E-01	1.7E-01
A_0069	Glucosamine 6-phosphate	440997	HMDB0001254	258.038	7.49	-5.3E-01	-3.0E-01
A_0070	*myo*-Inositol 2-phosphate	160886		259.022	8.80	-5.4E-01	3.3E-01
A_0071	Glucose 6-phosphate	5958	HMDB0001401	259.023	8.26	-5.4E-01	-3.2E-01

A_0072	*myo*-Inositol 1-phosphate<br>*myo*-Inositol 3-phosphate	107737<br>440194	HMDB0000213<br>HMDB0006814	259.023	8.61	4.8E-01	4.6E-01
A_0073	Glucose 1-phosphate	65533	HMDB0001586	259.023	8.48	-8.3E-01	-2.1E-01
A_0074	Fructose 6-phosphate	603	HMDB0000124	259.023	8.34	-4.7E-01	-4.1E-01
A_0075	Sorbitol 6-phosphate	152306	HMDB0005831	261.037	8.36	1.5E-01	4.8E-01
A_0076	2,3-Diphosphoglyceric acid	186004	HMDB0001294	264.953	14.19	8.0E-01	2.1E-01
A_0077	6-Phosphogluconic acid	91493	HMDB0001316	275.019	11.83	-9.7E-01	-9.1E-02
A_0078	Xanthosine	64959	HMDB0000299	283.070	6.47	-2.9E-01	8.3E-01
A_0079	Sedoheptulose 7-phosphate	165007	HMDB0001068	289.034	8.08	2.9E-01	-4.4E-01
A_0080	*N*-Acetylglucosamine 1-phosphate	440272	HMDB0001367	300.047	8.03	7.2E-01	2.6E-01
A_0081	*N*-Acetylglucosamine 6-phosphate	440996	HMDB0001062	300.048	7.72	-3.8E-01	-4.0E-01
A_0082	*N*-Acetylneuraminic acid	439197	HMDB0000230	308.100	6.14	3.4E-01	2.8E-01
A_0083	Ribulose 1,5-diphosphate	123658		308.979	12.45	5.6E-01	3.6E-01
A_0085	CMP	6131	HMDB0000095	322.043	8.04	3.5E-01	-1.6E-01
A_0086	UMP	6030	HMDB0000288	323.030	8.18	-7.5E-03	9.1E-01
A_0087	*N*-Glycolylneuraminic acid	440001	HMDB0000833	324.095	6.12	-7.9E-01	1.1E-01
A_0089	5-Aminoimidazole-4-carboxamide ribotide	65110	HMDB0001517	337.055	7.93	6.9E-01	3.9E-01
A_0090	Ascorbate 2-glucoside	54693473		337.075	6.09	-9.3E-01	3.6E-02
A_0091	Fructose 1,6-diphosphate	172313	HMDB0001058	338.989	11.73	-9.8E-01	-7.0E-02
A_0093	AMP	6083	HMDB0000045	346.056	7.78	-2.9E-01	9.0E-01
A_0094	3'-AMP	41211	HMDB0003540	346.058	8.16	-9.3E-01	-1.1E-01
A_0095	IMP	8582	HMDB0000175	347.042	7.98	-1.2E-01	8.5E-01
A_0096	GMP	6804	HMDB0001397	362.052	7.68	-1.9E-01	9.2E-01
A_0097	XA0055			368.999	11.56	-7.7E-01	-2.8E-01
A_0098	CoA_divalent	87642	HMDB0001423	382.549	8.82	7.6E-01	5.6E-01
A_0099	PRPP	7339	HMDB0000280	388.945	13.08	5.1E-01	1.7E-01
A_0100	FAD_divalent	643975	HMDB0001248	391.571	6.67	3.1E-01	6.6E-01
A_0103	UDP	6031	HMDB0000295	402.991	9.57	3.1E-01	-2.2E-01
A_0105	Cholic acid	221493	HMDB0000619	407.279	5.87	-8.1E-01	2.6E-01
A_0106	Thiamine diphosphate	1132	HMDB0001372	423.031	6.73	5.5E-01	-2.8E-01
A_0107	3-Methylcrotonyl CoA_divalent	9549326	HMDB0001493	423.570	8.60	-2.7E-01	3.8E-01
A_0109	ADP	6022	HMDB0001341	426.025	9.01	6.4E-01	6.6E-01
A_0110	GDP	8977	HMDB0001201	442.015	8.81	2.2E-01	8.5E-01
A_0111	XA0065			445.054	5.92	2.4E-01	7.7E-01
A_0112	Adenylosuccinic acid	447145	HMDB0000536	462.069	11.15	-9.6E-01	2.2E-01
A_0117	CDP-choline	13804	HMDB0001413	487.100	5.80	4.7E-01	7.3E-01
A_0119	ATP	5957	HMDB0000538	505.987	9.64	9.2E-01	-3.4E-01
A_0120	ITP	8583	HMDB0000189	506.972	9.65	5.5E-01	7.3E-01
A_0121	GTP	6830	HMDB0001273	521.976	9.42	9.2E-01	-3.6E-01
A_0122	ADP-ribose	445794	HMDB0001178	558.068	7.18	-8.8E-01	2.8E-01
A_0123	UDP-galactose<br>UDP-glucose	23724458<br>8629	HMDB0000302<br>HMDB0000286	565.044	7.28	-9.7E-01	9.7E-02
A_0124	UDP-glucuronic acid	17473	HMDB0000935	579.030	9.15	8.0E-01	1.1E-01
A_0125	GDP-fucose<br>ADP-glucose	10918995<br>16500	HMDB0001095<br>HMDB0006557	588.075	7.02	-4.5E-01	5.0E-01
A_0126	UDP-*N*-acetylgalactosamine<br>UDP-*N*-acetylglucosamine	23724461<br>445675	HMDB0000304<br>HMDB0000290	606.069	7.13	-9.6E-01	-2.7E-02
A_0127	CMP-*N*-acetylneuraminate	448209	HMDB0001176	613.143	7.03	-5.7E-01	-5.5E-01
A_0128	NAD^+	5893	HMDB0000902	662.105	5.59	9.6E-01	2.7E-01
A_0129	NADH	439153	HMDB0001487	664.111	6.91	9.1E-01	-1.3E-01
A_0130	$NADP^+$	5886	HMDB0000217	742.067	7.85	8.8E-01	1.8E-01
C_0001	Trimethylamine	1146	HMDB0000906	60.081	4.82	-8.7E-01	-3.0E-01
C_0002	Urea	1176	HMDB0000294	61.040	17.31	-7.4E-02	-3.6E-01
C_0003	Ethanolamine	700	HMDB0000149	62.061	5.20	9.0E-01	2.5E-01
C_0004	XC0001			72.081	5.21	-9.9E-01	-7.8E-02
C_0005	Aminoacetone	215	HMDB0002134	74.060	5.60	-2.0E-01	1.9E-02
C_0006	Gly	750	HMDB0000123	76.040	6.80	-9.3E-01	-2.8E-03
C_0007	Isopropanolamine	4	HMDB0012136	76.075	5.71	-4.8E-01	-5.2E-03
C_0008	Trimethylamine *N*-oxide	1145	HMDB0000925	76.076	5.40	-9.3E-01	5.4E-02
C_0009	Putrescine	1045	HMDB0001414	89.108	3.87	-3.3E-02	6.2E-01
C_0010	β-Ala	239	HMDB0000056	90.055	5.98	9.6E-01	1.2E-01
C_0011	Sarcosine	1088	HMDB0000271	90.055	7.75	5.8E-01	3.5E-01
C_0012	Ala	602	HMDB0000161,HMDB0001310	90.055	7.35	-9.5E-01	-1.4E-01
C_0013	Dimethylaminoethanol	7902	HMDB0032231	90.092	5.64	-2.3E-01	2.2E-01
C_0014	Glycerol	753	HMDB0000131	93.055	18.05	5.5E-01	-4.1E-01
C_0015	Phenol	996	HMDB0000228	95.048	4.41	5.3E-01	-5.8E-01
C_0016	Homoserinelactone	73509		102.055	5.77	3.0E-01	9.1E-02
C_0017	Azetidine 2-carboxylic acid	16486		102.055	9.40	-4.8E-01	-5.2E-03
C_0018	Hexylamine	8102		102.127	6.18	-8.0E-01	3.6E-01
C_0019	3-Aminoisobutyric acid	64956	HMDB0003911	104.071	6.40	-9.3E-01	-2.5E-01
C_0020	GABA	119	HMDB0000112	104.071	6.27	-8.6E-01	4.1E-01

C_0021	3-Aminobutyric acid	10932		104.071	6.48	7.7E-01	-5.7E-01
C_0022	2-Aminoisobutyric acid<br>2-Aminobutyric acid	6119<br>6657	HMDB0001906<br>HMDB0000452	104.071	7.84	-5.0E-01	3.0E-01
C_0023	*N*,*N*-Dimethylglycine	673	HMDB0000092	104.071	8.91	2.8E-01	3.7E-01
C_0024	Choline	305	HMDB0000097	104.107	5.58	8.1E-01	2.1E-01
C_0025	2,3-Diaminopropionic acid	364	HMDB0002006	105.067	5.86	-7.9E-01	-2.3E-01
C_0026	Ser	617	HMDB0000187,HMDB0003406	106.050	8.17	-8.7E-01	-2.8E-01
C_0027	Diethanolamine	8113	HMDB0004437	106.087	6.23	6.0E-02	2.8E-01
C_0028	Hypotaurine	107812	HMDB0000965	110.027	14.56	-5.1E-01	-7.5E-01
C_0029	Cytosine	597	HMDB0000630	112.052	5.92	-9.9E-01	2.3E-02
C_0030	Histamine	774	HMDB0000870	112.088	3.94	4.2E-01	5.0E-01
C_0031	Uracil	1174	HMDB0000300	113.035	18.10	3.9E-01	-7.4E-01
C_0032	Creatinine	588	HMDB0000562	114.067	5.94	3.9E-01	-1.4E-01
C_0033	3-Amino-2-piperidone	5200225	HMDB0000323	115.086	6.20	-3.2E-01	4.4E-01
C_0034	Pro	614	HMDB0000162,HMDB0003411	116.071	8.79	1.4E-01	3.4E-01
C_0035	Guanidoacetic acid	763	HMDB0000128	118.062	6.72	-5.5E-01	5.8E-01
C_0036	Val	1182	HMDB0000883	118.087	8.13	3.0E-01	9.1E-01
C_0037	Betaine	247	HMDB0000043	118.087	9.17	8.0E-01	-4.8E-01
C_0038	5-Aminovaleric acid	138	HMDB0003355	118.087	6.52	-9.6E-01	6.5E-02
C_0039	2,4-Diaminobutyric acid	134490	HMDB0006284	119.082	5.65	-9.4E-01	-9.5E-02
C_0040	4-Amino-3-hydroxybutyric acid	2149		120.064	6.61	-5.5E-01	-2.1E-01
C_0041	Thr	6288	HMDB0000167	120.066	8.57	-7.9E-01	1.2E-01
C_0042	Homoserine	12647	HMDB0000719	120.066	8.22	-8.9E-01	-1.5E-01
C_0043	2-Methylserine	439656		120.067	8.45	-7.6E-01	-3.6E-01
C_0044	Betaine aldehyde_+H_2O	249	HMDB0001252	120.103	6.06	8.2E-01	-1.4E-01
C_0045	Anserine_divalent	112072	HMDB0000194	121.069	5.56	-4.6E-01	3.5E-01
C_0046	Cys	594	HMDB0000574,HMDB0003417	122.028	9.22	-9.6E-01	2.7E-01
C_0047	2-Amino-2-(hydroxymethyl)-1,3-propanediol	6503		122.082	6.74	-6.6E-01	-1.0E-01
C_0048	Nicotinamide	936	HMDB0001406	123.056	6.04	-6.4E-01	-3.8E-01
C_0049	Nicotinic acid	938	HMDB0001488	124.040	8.19	-3.1E-01	-6.8E-01
C_0050	Picolinic acid	1018	HMDB0002243	124.040	15.28	3.3E-01	-6.5E-01
C_0051	Taurine	1123	HMDB0000251	126.022	18.03	8.9E-01	2.2E-01
C_0052	1-Methylhistamine	3614	HMDB0000898	126.103	4.04	3.7E-01	4.9E-01
C_0053	3-Hydroxy-2-methyl-4-pyrone	8369	HMDB0030776	127.038	18.07	-9.9E-01	-6.0E-02
C_0055	Imidazole-4-acetic acid	96215	HMDB0002024	127.051	6.54	-1.5E-01	-1.6E-01
C_0056	XC0016			129.067	7.13	-7.1E-01	3.2E-01
C_0057	4-Oxopyrrolidine-2-carboxylic acid	107541		130.050	8.92	6.1E-01	3.0E-02
C_0058	Pipecolic acid	439227	HMDB0000070,HMDB0000716,HMDB0005960	130.087	8.34	-5.8E-01	-5.8E-01
C_0059	*trans*-Glutaconic acid	5280498	HMDB0000620	131.034	18.78	-7.2E-01	2.8E-01
C_0060	*cis*-4-Hydroxyproline	440014	HMDB0006055	132.066	8.97	5.8E-01	-5.8E-01
C_0061	Hydroxyproline	5810	HMDB0000725	132.067	9.79	-8.9E-01	-1.6E-01
C_0062	3-Guanidinopropionic acid	67701		132.077	6.53	-9.0E-01	-1.9E-01
C_0063	Creatine	586	HMDB0000064	132.078	7.20	-1.8E-02	-5.5E-02
C_0064	Ile	791	HMDB0000172	132.102	8.28	7.4E-02	9.5E-01
C_0065	Leu	857	HMDB0000687	132.102	8.37	1.6E-01	9.4E-01
C_0066	Asn	236	HMDB0000168,HMDB0033780	133.061	8.58	-8.0E-01	5.4E-01
C_0067	Gly-Gly	11163	HMDB0011733	133.062	6.80	6.6E-01	7.0E-01
C_0068	Ornithine	389	HMDB0000214,HMDB0003374	133.098	5.55	6.6E-01	-5.2E-01
C_0069	Thiaproline	9934		134.028	11.38	4.0E-01	6.9E-01
C_0070	Asp	424	HMDB0000191,HMDB0006483	134.045	9.47	-8.9E-01	-7.9E-02
C_0071	Adenine	190	HMDB0000034	136.063	6.22	2.6E-01	-8.8E-01
C_0072	Hypoxanthine	790	HMDB0000157	137.047	9.27	6.8E-01	-6.7E-01
C_0073	1-Methylnicotinamide	457	HMDB0000699	137.072	5.99	-8.1E-01	4.9E-01
C_0074	Trigonelline	5570	HMDB0000875	138.056	8.53	-5.7E-01	-2.3E-01
C_0075	Anthranilic acid	227	HMDB0001123	138.056	8.87	6.7E-01	7.2E-02
C_0077	γ-Glu-Lys_divalent	65254	HMDB0029154	138.581	6.99	-1.5E-01	6.8E-02
C_0078	Urocanic acid	736715	HMDB0000301	139.051	6.76	-7.5E-02	5.9E-01
C_0079	1-Methyl-4-imidazoleacetic acid	75810	HMDB0002820	141.066	6.71	-8.5E-02	-2.9E-01
C_0080	1*H*-Imidazole-4-propionic acid	10105257		141.067	6.61	-7.4E-01	-9.0E-02
C_0081	Ectoine	126041		143.082	7.65	5.4E-02	-3.6E-01
C_0082	XC0029<br>Stachydrine	0<br>115244	 HMDB0004827	144.102	9.32	3.8E-01	-4.6E-02
C_0083	4-Guanidinobutyric acid	500	HMDB0003464	146.093	6.75	-7.2E-01	7.1E-02
C_0084	γ-Butyrobetaine	134	HMDB0001161	146.118	6.60	-9.4E-01	-6.9E-02
C_0085	Spermidine	1102	HMDB0001257	146.166	3.72	-4.7E-01	-2.5E-01
C_0086	Gln	738	HMDB0000641,HMDB0003423	147.077	8.76	-8.9E-01	3.5E-01
C_0087	Lys	866	HMDB0000182,HMDB0003405	147.113	5.60	-2.8E-01	5.7E-02
C_0088	Isoglutamic acid	73064		148.061	7.45	8.4E-01	-2.6E-01
C_0089	Glu	611	HMDB0000148,HMDB0003339	148.061	8.92	7.5E-01	-1.1E-01
C_0090	*threo*-β-Methylaspartic acid	440064		148.062	10.05	-1.1E-01	1.7E-02
C_0091	Met	876	HMDB0000696	150.059	8.73	7.5E-01	1.2E-01

C_0092	Guanine	764	HMDB0000132	152.059	6.79	7.8E-01	-4.6E-01
C_0093	Xanthine	1188	HMDB0000292	153.042	16.04	9.4E-01	2.0E-01
C_0094	N^1-Methyl-4-pyridone-5-carboxamide	440810	HMDB0004194	153.066	15.08	2.6E-01	6.2E-01
C_0095	4-(β-Acetylaminoethyl)imidazole	69602		154.097	6.85	1.4E-01	3.8E-01
C_0096	His	773	HMDB0000177	156.078	5.95	-9.3E-01	6.5E-02
C_0097	Imidazolelactic acid	793		157.061	7.18	-5.1E-01	2.5E-01
C_0098	XC0145<br>Ala-Ala	15331<br>5460362	 HMDB0003459	161.093	7.52	-6.4E-01	5.4E-01
C_0099	Tryptamine	1150	HMDB0000303	161.106	6.76	4.0E-01	-8.0E-01
C_0100	N^6-Methyllysine	164795	HMDB0002038	161.129	5.79	-9.4E-01	-1.0E-01
C_0101	*O*-Acetylhomoserine<br>2-Aminoadipic acid	439389<br>92136	 HMDB0000510	162.077	8.92	1.6E-01	2.1E-01
C_0102	Carnitine	85	HMDB0000062	162.113	6.92	-1.0E-01	-9.6E-01
C_0103	5-Hydroxylysine	3032849	HMDB0000450	163.109	5.83	1.4E-01	5.4E-01
C_0104	Pterin	73000	HMDB0000802	164.057	8.49	8.6E-01	1.5E-01
C_0105	Phe	994	HMDB0000159	166.087	9.00	2.4E-02	7.7E-01
C_0106	Tyr-Arg_divalent	123804		169.595	6.27	5.1E-01	4.8E-01
C_0107	1-Methylhistidine<br>3-Methylhistidine	92105<br>64969	HMDB0000001<br>HMDB0000479	170.094	6.12	-7.3E-01	-1.1E-01
C_0108	XC0147	4173		172.072	7.85	4.9E-01	5.5E-01
C_0109	XC0040			174.088	9.79	-1.2E-01	-7.8E-01
C_0110	N^5-Ethylglutamine	439378		175.109	9.24	-7.6E-01	-8.6E-02
C_0111	*N*-Acetylornithine	439232	HMDB0003357	175.109	7.73	-1.4E-01	4.9E-01
C_0112	Arg	6322	HMDB0000517,HMDB0003416	175.120	5.81	-9.7E-01	-4.4E-02
C_0113	Guanidinosuccinic acid	439918	HMDB0003157	176.068	8.23	-9.7E-01	1.1E-01
C_0114	Citrulline	9750	HMDB0000904	176.104	8.99	8.8E-01	-3.6E-01
C_0115	Serotonin	5202	HMDB0000259	177.103	7.13	3.9E-01	-2.0E-01
C_0116	Gluconolactone	7027	HMDB0000150	179.056	18.76	8.8E-01	3.2E-01
C_0117	Glucosamine	439213	HMDB0001514	180.088	7.54	-9.2E-01	-3.2E-01
C_0118	Tyr	1153	HMDB0000158	182.082	9.22	-5.9E-01	6.5E-01
C_0119	Phosphorylcholine	1014	HMDB0001565	184.074	16.66	-4.3E-01	-3.6E-01
C_0120	N^1-Acetylspermidine	496	HMDB0001276	188.177	5.16	-4.1E-01	2.4E-01
C_0121	Gly-Leu			189.124	7.83	3.9E-01	7.3E-01
C_0122	*N*-Acetyllysine	92907	HMDB0000446	189.125	7.93	-2.6E-01	4.1E-01
C_0123	N^6-Acetyllysine	92832	HMDB0000206	189.125	9.29	5.0E-01	-2.3E-01
C_0124	N_{ω}-Methylarginine	132862		189.136	6.07	-3.5E-01	-4.0E-01
C_0125	N^6,N^6,N^6-Trimethyllysine	440120	HMDB0001325	189.159	5.86	8.3E-01	1.6E-01
C_0126	Homocitrulline	65072	HMDB0000679	190.120	9.08	-8.8E-01	2.4E-01
C_0127	Gly-Asp	97363		191.067	8.04	-4.4E-01	8.0E-01
C_0128	2,6-Diaminopimelic acid	439283	HMDB0001370	191.103	7.24	-3.7E-01	-5.7E-01
C_0129	*N*-Acetylhistidine	75619		198.089	7.97	-7.3E-01	1.3E-01
C_0130	11-Aminoundecanoic acid	17083		202.182	7.81	4.9E-01	7.7E-01
C_0131	SDMA	169148	HMDB0003334	203.151	6.35	8.1E-01	-4.0E-01
C_0132	ADMA	123831	HMDB0001539	203.151	6.24	2.0E-01	-9.2E-01
C_0133	Spermine	1103	HMDB0001256	203.225	3.67	9.7E-01	-1.4E-01
C_0134	*O*-Acetylcarnitine	439756	HMDB0000201	204.124	7.33	-9.3E-02	-7.5E-01
C_0135	γ-Glu-Gly	165527	HMDB0011667	205.083	9.81	-9.6E-01	-1.4E-01
C_0136	Trp	1148	HMDB0000929	205.098	8.95	-7.5E-01	4.7E-01
C_0137	Carboxymethyllysine	123800		205.120	7.52	9.3E-01	1.5E-01
C_0138	Kynurenine	846	HMDB0000684	209.094	8.17	1.5E-01	7.9E-02
C_0139	Propionylcarnitine<br>XC0061	188824<br>0	HMDB0000824	218.140	7.57	4.4E-01	-6.0E-01
C_0140	β-Ala-Lys	440638		218.151	5.54	-8.2E-01	4.5E-01
C_0141	XC0065			221.093	10.63	-6.3E-01	-4.3E-01
C_0142	*N*-Acetylglucosylamine	439454	HMDB0001104	221.114	8.05	8.7E-01	-2.6E-01
C_0143	*N*-Acetylgalactosamine<br>*N*-Acetylglucosamine<br>*N*-Acetylmannosamine	35717<br>439174<br>439281	HMDB0000853<br>HMDB0000215<br>HMDB0001129	222.099	18.09	1.4E-01	-6.7E-01
C_0144	Carnosine	439224	HMDB0000033	227.115	5.51	3.3E-01	-4.1E-01
C_0145	Ergothioneine	3037043	HMDB0003045	230.097	14.04	7.8E-01	-4.5E-01
C_0146	Butyrylcarnitine	439829	HMDB0002013	232.156	7.77	-4.4E-01	3.9E-01
C_0147	Ser-Glu			235.094	8.43	-7.1E-01	4.6E-01
C_0148	γ-Glu-Ser	22844748	HMDB0029158	235.094	10.25	-7.8E-01	1.3E-01
C_0149	Thr-Asp	3280446		235.095	8.61	-6.4E-01	5.1E-01
C_0150	7,8-Dihydrobiopterin	119055	HMDB0000038	240.110	8.96	2.8E-01	-5.1E-02
C_0151	Thymidine	5789	HMDB0000273	243.098	18.18	-7.1E-01	1.7E-01
C_0152	Cytidine	6175	HMDB0000089	244.095	7.86	6.8E-01	-7.0E-01
C_0153	Uridine	6029	HMDB0000296	245.078	18.11	-9.3E-01	-2.6E-01
C_0154	Isovalerylcarnitine	6426851	HMDB0000688	246.170	7.87	8.4E-01	2.8E-01
C_0155	γ-Glu-Val	7015683	HMDB0011172	247.131	10.33	-1.9E-02	-6.0E-01
C_0156	Malonylcarnitine	22833583	HMDB0002095	248.114	8.28	5.7E-01	3.0E-01

C_0157	Pyridoxamine 5'-phosphate	1053	HMDB0001555	249.065	8.51	6.4E-01	-8.9E-02
C_0158	γ-Glu-Thr	53861142	HMDB0029159	249.110	10.34	-1.8E-01	-8.0E-01
C_0159	γ-Glu-Cys	123938	HMDB0001049	251.071	10.43	-7.0E-01	5.7E-01
C_0160	XC0153	4725		254.123	8.45	-5.5E-01	8.1E-01
C_0161	XC0089			255.100	7.72	9.2E-01	-3.2E-01
C_0162	XC0154	3182		255.110	18.12	-9.4E-01	-2.7E-02
C_0163	Glycerophosphocholine	439285	HMDB0000086	258.112	17.68	-9.2E-01	-4.1E-02
C_0164	γ-Glu-Ile<br>γ-Glu-Leu	22885096<br>151023	HMDB0011170<br>HMDB0011171	261.146	10.49	-1.1E-01	-5.1E-02
C_0165	γ-Glu-Asn	131801686	HMDB0029144	262.103	10.37	4.4E-01	5.6E-02
C_0166	γ-Glu-Ornitine	189156	HMDB0002248	262.141	6.93	3.2E-01	8.2E-01
C_0167	γ-Glu-Asp	161197	HMDB0030419	263.087	10.57	7.1E-02	9.0E-01
C_0168	Thiamine	1130	HMDB0000235	265.113	5.36	-9.6E-01	-1.6E-01
C_0169	Adenosine	60961	HMDB0000050	268.105	8.03	6.6E-01	-4.0E-01
C_0170	Inosine	6021	HMDB0000195	269.089	16.01	2.5E-02	-9.3E-01
C_0171	γ-Glu-Glu	92865	HMDB0011737	277.105	10.66	2.7E-01	-7.9E-01
C_0172	Glu-Glu	439500		277.106	8.85	-2.5E-01	9.3E-01
C_0173	Saccharopine	160556	HMDB0000279	277.141	8.74	8.7E-01	3.1E-01
C_0174	1-Methyladenosine	27476	HMDB0003331	282.122	8.08	5.7E-01	4.4E-01
C_0175	Guanosine	6802	HMDB0000133	284.101	10.31	6.0E-01	-7.7E-01
C_0176	His-Glu	7010583		285.121	6.16	-7.8E-01	1.5E-01
C_0177	Ophthalmic acid	7018721	HMDB0005765	290.135	10.73	-7.1E-01	-3.4E-01
C_0178	Argininosuccinic acid	16950	HMDB0000052	291.131	7.69	-9.8E-01	1.1E-01
C_0179	γ-Glu-Phe	111299	HMDB0000594	295.130	10.59	-3.0E-02	-6.3E-01
C_0180	5'-Deoxy-5'-methylthioadenosine	439176	HMDB0001173	298.098	8.18	-5.0E-01	-2.4E-01
C_0181	Arg-Glu			304.163	6.11	-7.1E-01	3.9E-01
C_0182	Glutathione (GSSG)_divalent	65359	HMDB0003337	307.085	9.91	-4.2E-01	-5.2E-01
C_0183	Glutathione (GSH)	124886	HMDB0000125	308.093	10.75	-9.6E-01	-1.0E-01
C_0184	XC0126			310.114	12.18	5.4E-01	-2.9E-01
C_0185	Tyr-Glu			311.125	9.03	-5.8E-01	3.7E-01
C_0186	*S*-Methylglutathione	115260		322.108	10.86	-9.1E-01	-4.9E-02
C_0187	XC0132			325.162	7.06	3.3E-01	9.8E-02
C_0188	NMN	14180	HMDB0000229	335.066	16.77	9.6E-01	-4.2E-02
C_0189	Thiamine phosphate	1131	HMDB0002666	345.080	8.67	6.9E-01	3.9E-01
C_0190	XC0137			350.103	10.98	2.4E-01	-4.0E-01
C_0191	*S*-Lactoylglutathione	440018	HMDB0001066	380.114	11.20	7.7E-01	-4.5E-01
C_0192	*S*-Adenosylhomocysteine	439155	HMDB0000939	385.130	7.13	8.4E-01	4.5E-01
C_0193	*S*-Adenosylmethionine	34755	HMDB0001185	399.146	5.81	-9.1E-01	9.7E-02
C_0194	Tetrahydrofolic acid	135444742	HMDB0001846	446.181	9.65	-1.3E-01	-4.3E-01
C_0195	5-Methyltetrahydrofolic acid	444412	HMDB0001396	460.196	9.63	-5.0E-01	-1.8E-01

MT, migration time; PC, principal component; RT, retention time
