# Supplementary material for: A Non-Obese Hyperglycemic Mouse Model that Develops after Birth with Low Birthweight
Source: Biomedicines. 2022 Jul 8;10(7):1642. doi: 10.3390/biomedicines10071642 (PMC9312481; doi:10.3390/biomedicines10071642)
Supplement: Supplementary file 1 [file biomedicines-10-01642-s001.zip › Supplementary Table 3.pdf]

Supplementary Table S3. Results of comparative analyses

ID	Metabolite	PubChem CID	HMDB ID	Concentration (nmol/g)										Comparative Analysis		
				Group I			Group C			Group I		Group C		Group I vs Group C		
				I1	I2	I3	C5	C6	C7	Mean	S.D.	Mean	S.D.	Ratio¶	*p*-value‖	
A_0007	2-Hydroxybutyric acid	440864	HMDB0000008	23	N.D.	42	68	74	27	32	14	56	26	0.6	0.267	
A_0021	2-Oxoglutaric acid	51	HMDB0000208	N.D.	N.D.	N.D.	N.D.	N.D.	N.D.	N.A.	N.A.	N.A.	N.A.	N.A.	N.A.	
A_0009	2-Oxoisovaleric acid	49	HMDB0000019	N.D.	N.D.	N.D.	N.D.	N.D.	N.D.	N.A.	N.A.	N.A.	N.A.	N.A.	N.A.	
A_0041	2-Phosphoglyceric acid	439278	HMDB0003391	N.D.	N.D.	N.D.	N.D.	N.D.	N.D.	N.A.	N.A.	N.A.	N.A.	N.A.	N.A.	
A_0006	3-Hydroxybutyric acid	441	HMDB0000011,HMDB0000357,HMDB0000442	240	322	413	727	1,008	499	325	87	744	255	0.4	0.092	
A_0040	3-Phosphoglyceric acid	439183	HMDB0000807	N.D.	N.D.	28	17	28	28	28	N.A.	25	6.3	1.1	N.A.	
A_0077	6-Phosphogluconic acid	91493	HMDB0001316	110	99	81	37	48	44	97	15	43	5.6	2.2	0.015	*
A_0104	Acetyl CoA_divalent	444493		N.D.	N.D.	N.D.	N.D.	N.D.	N.D.	N.A.	N.A.	N.A.	N.A.	N.A.	N.A.	
C_0071	Adenine	190	HMDB0000034	8.1	7.1	6.9	7.2	6.8	11	7.4	0.6	8.3	2.2	0.9	0.537	
C_0169	Adenosine	60961	HMDB0000050	69	49	47	63	83	73	55	12	73	10	0.8	0.123	
A_0109	ADP	6022	HMDB0001341	106	99	115	141	146	99	107	7.9	129	26	0.8	0.278	
C_0012	Ala	602	HMDB0000161,HMDB0001310	2,722	2,240	2,372	1,435	1,167	1,651	2,445	249	1,418	243	1.7	0.007	**
A_0093	AMP	6083	HMDB0000045	788	748	889	894	791	331	808	72	672	299	1.2	0.516	
C_0075	Anthranilic acid	227	HMDB0001123	N.D.	N.D.	0.4	0.3	0.3	0.4	0.4	N.A.	0.3	0.07	1.2	N.A.	
C_0112	Arg	6322	HMDB0000517,HMDB0003416	8.7	7.6	5.8	3.5	2.8	2.9	7.4	1.4	3.1	0.4	2.4	0.030	*
C_0066	Asn	236	HMDB0000168,HMDB0033780	230	215	246	200	168	142	230	16	170	29	1.4	0.050	
C_0070	Asp	424	HMDB0000191,HMDB0006483	576	592	519	366	473	413	562	38	417	54	1.3	0.023	*
A_0119	ATP	5957	HMDB0000538	15	18	16	28	37	40	16	1.6	35	6.5	0.5	0.031	*
C_0037	Betaine	247	HMDB0000043	1,077	948	1,092	1,155	1,520	1,624	1,039	79	1,433	247	0.7	0.098	
C_0044	Betaine aldehyde_+H_2O	249		52	53	64	64	99	84	56	7.1	82	17	0.7	0.107	
A_0088	cAMP	6076	HMDB0000058	N.D.	N.D.	N.D.	N.D.	N.D.	N.D.	N.A.	N.A.	N.A.	N.A.	N.A.	N.A.	
C_0144	Carnosine	439224	HMDB0000033	0.4	0.8	0.6	0.8	0.5	1.0	0.6	0.2	0.8	0.3	0.8	0.451	
A_0102	CDP	6132	HMDB0001546	N.D.	N.D.	N.D.	N.D.	N.D.	N.D.	N.A.	N.A.	N.A.	N.A.	N.A.	N.A.	
A_0092	cGMP	24316	HMDB0001314	N.D.	N.D.	N.D.	N.D.	N.D.	N.D.	N.A.	N.A.	N.A.	N.A.	N.A.	N.A.	
C_0024	Choline	305	HMDB0000097	398	417	488	581	857	552	434	47	664	168	0.7	0.134	
A_0034	*cis*-Aconitic acid	643757	HMDB0000072	N.D.	N.D.	N.D.	N.D.	N.D.	N.D.	N.A.	N.A.	N.A.	N.A.	N.A.	N.A.	
A_0049	Citric acid	311	HMDB0000094	187	225	182	137	148	187	198	24	157	26	1.3	0.118	
C_0114	Citrulline	9750	HMDB0000904	33	30	34	36	40	41	32	1.8	39	2.7	0.8	0.028	*
A_0085	CMP	6131	HMDB0000095	78	60	64	70	78	69	68	9.4	73	4.8	0.9	0.474	
A_0098	CoA_divalent	87642		105	98	130	142	143	117	111	17	134	15	0.8	0.147	
C_0063	Creatine	586	HMDB0000064	193	165	244	211	152	230	201	40	198	41	1.0	0.931	
C_0032	Creatinine	588	HMDB0000562	4.4	4.3	5.5	5.4	4.3	5.9	4.7	0.7	5.2	0.8	0.9	0.471	
A_0115	CTP	6176	HMDB0000082	N.D.	N.D.	N.D.	N.D.	N.D.	N.D.	N.A.	N.A.	N.A.	N.A.	N.A.	N.A.	
C_0046	Cys	594	HMDB0000574,HMDB0003417	8.3	7.8	7.5	3.8	2.6	1.4	7.9	0.4	2.6	1.2	3.0	0.011	*
C_0152	Cytidine	6175	HMDB0000089	6.8	4.4	4.9	6.6	8.2	11	5.4	1.2	8.8	2.5	0.6	0.125	
C_0029	Cytosine	597	HMDB0000630	0.6	0.5	0.5	N.D.	N.D.	N.D.	0.5	0.08	N.A.	N.A.	1<	N.A.	
A_0118	dATP	15993	HMDB0001532	N.D.	N.D.	N.D.	N.D.	N.D.	N.D.	N.A.	N.A.	N.A.	N.A.	N.A.	N.A.	
A_0113	dCTP	65091	HMDB0000998	N.D.	N.D.	N.D.	N.D.	N.D.	N.D.	N.A.	N.A.	N.A.	N.A.	N.A.	N.A.	
A_0029	Dihydroxyacetone phosphate	668	HMDB0001473	698	522	436	134	189	150	552	133	158	28	3.5	0.032	*
A_0101	dTDP	164628	HMDB0001274	N.D.	N.D.	N.D.	N.D.	N.D.	N.D.	N.A.	N.A.	N.A.	N.A.	N.A.	N.A.	
A_0084	dTMP	9700	HMDB0001227	N.D.	N.D.	N.D.	N.D.	N.D.	N.D.	N.A.	N.A.	N.A.	N.A.	N.A.	N.A.	
A_0114	dTTP	64968	HMDB0001342	N.D.	N.D.	N.D.	N.D.	N.D.	N.D.	N.A.	N.A.	N.A.	N.A.	N.A.	N.A.	
A_0056	Erythrose 4-phosphate	122357	HMDB0001321	N.D.	N.D.	N.D.	N.D.	N.D.	N.D.	N.A.	N.A.	N.A.	N.A.	N.A.	N.A.	
A_0091	Fructose 1,6-diphosphate	172313	HMDB0001058	412	313	257	30	20	42	327	78	31	11	11	0.021	*
A_0074	Fructose 6-phosphate	603	HMDB0000124	419	95	59	80	98	116	191	198	98	18	1.9	0.503	
A_0008	Fumaric acid	444972	HMDB0000134	860	792	835	261	331	332	829	34	308	41	2.7	8.9E-05	***
C_0020	GABA	119	HMDB0000112	38	41	49	24	27	18	43	5.5	23	4.5	1.9	0.009	**
A_0110	GDP	8977	HMDB0001201	21	21	26	24	25	20	23	2.8	23	2.3	1.0	0.875	
C_0086	Gln	738	HMDB0000641,HMDB0003423	2,245	2,531	2,173	1,737	1,667	1,272	2,316	189	1,559	251	1.5	0.016	*
C_0089	Glu	611	HMDB0000148,HMDB0003339	1,662	1,528	1,422	1,735	2,099	1,743	1,537	120	1,859	208	0.8	0.098	
A_0055	Gluconic acid	10690	HMDB0000625	629	483	602	1,014	1,045	734	571	78	931	172	0.6	0.051	
A_0073	Glucose 1-phosphate	65533	HMDB0001586	222	106	72	25	20	25	133	79	23	3.2	5.7	0.136	
A_0071	Glucose 6-phosphate	5958	HMDB0001401	1,726	234	126	149	163	155	695	894	156	6.9	4.5	0.405	
C_0183	Glutathione (GSH)	124886	HMDB0000125	4,479	3,705	3,391	2,212	2,224	2,287	3,858	560	2,241	40	1.7	0.037	*
C_0182	Glutathione (GSSG)_divalent	65359		671	563	540	540	571	574	591	70	561	19	1.1	0.543	
C_0006	Gly	750	HMDB0000123	1,980	1,679	1,781	1,396	1,404	1,388	1,814	153	1,396	7.7	1.3	0.042	*
A_0030	Glyceraldehyde 3-phosphate	729	HMDB0001112	36	N.D.	N.D.	N.D.	N.D.	N.D.	36	N.A.	N.A.	N.A.	1<	N.A.	
A_0031	Glycerol 3-phosphate	439162	HMDB0000126	2,125	2,306	2,255	1,994	2,526	1,990	2,229	93	2,170	308	1.0	0.778	
A_0002	Glycolic acid	757	HMDB0000115	N.D.	N.D.	N.D.	N.D.	N.D.	N.D.	N.A.	N.A.	N.A.	N.A.	N.A.	N.A.	
A_0001	Glyoxylic acid	760	HMDB0000119	N.D.	N.D.	N.D.	N.D.	N.D.	N.D.	N.A.	N.A.	N.A.	N.A.	N.A.	N.A.	
A_0096	GMP	6804	HMDB0001397	245	230	300	281	276	116	259	37	225	94	1.2	0.606	
A_0121	GTP	6830	HMDB0001273	N.D.	N.D.	N.D.	7.1	12	14	N.A.	N.A.	11	3.8	<1	N.A.	
C_0092	Guanine	764	HMDB0000132	2.9	2.7	3.3	3.3	5.3	5.6	3.0	0.3	4.7	1.2	0.6	0.130	
C_0175	Guanosine	6802	HMDB0000133	57	47	46	51	66	82	50	6.3	66	16	0.8	0.199	
C_0096	His	773	HMDB0000177	693	644	618	571	556	535	652	38	554	18	1.2	0.031	*
C_0042	Homoserine	12647	HMDB0000719	2.1	1.8	2.2	0.8	1.0	1.4	2.0	0.2	1.1	0.3	1.9	0.014	*
C_0061	Hydroxyproline	5810	HMDB0000725	22	19	24	9.6	10	16	22	2.3	12	3.3	1.8	0.017	*
C_0072	Hypoxanthine	790	HMDB0000157	821	721	792	821	972	1,175	778	52	989	178	0.8	0.168	
C_0064	Ile	791	HMDB0000172	280	283	317	317	319	239	293	20	292	46	1.0	0.956	
A_0095	IMP	8582	HMDB0000175	217	207	216	262	252	61	214	5.8	191	113	1.1	0.767	
C_0170	Inosine	6021	HMDB0000195	2,078	1,789	1,834	1,708	1,856	2,241	1,901	155	1,935	275	1.0	0.862	
A_0047	Isocitric acid	1198	HMDB0000193	N.D.	N.D.	N.D.	N.D.	N.D.	N.D.	N.A.	N.A.	N.A.	N.A.	N.A.	N.A.	
A_0005	Lactic acid	612	HMDB0000190,HMDB0001311	14,946	14,349	17,523	5,675	3,948	5,925	15,606	1,687	5,183	1,077	3.0	0.002	**
C_0065	Leu	857	HMDB0000687	543	539	621	614	589	511	568	46	572	54	1.0	0.932	
C_0087	Lys	866	HMDB0000182,HMDB0003405	1,130	1,035	1,249	1,171	902	1,152	1,138	107	1,075	150	1.1	0.588	
A_0016	Malic acid	525	HMDB0000156,HMDB0000744	2,245	2,164	2,154	470	619	651	2,188	50	580	96	3.8	1.3E-04	***
A_0108	Malonyl CoA_divalent	644066		N.D.	N.D.	N.D.	N.D.	N.D.	N.D.	N.A.	N.A.	N.A.	N.A.	N.A.	N.A.	
C_0091	Met	876	HMDB0000696	202	176	207	222	212	214	195	16	216	5.3	0.9	0.144	
C_0023	*N*,*N*-Dimethylglycine	673	HMDB0000092	43	29	47	46	41	39	40	9.6	42	3.7	0.9	0.683	
A_0128	NAD^+	5893	HMDB0000902	26	30	37	65	63	50	31	5.5	60	8.1	0.5	0.010	*
A_0130	$NADP^+$	5886	HMDB0000217	16	13	11	32	30	21	13	2.5	28	5.7	0.5	0.031	*
C_0068	Ornithine	389	HMDB0000214,HMDB0003374	363	370	417	384	464	532	383	30	460	74	0.8	0.207	
C_0105	Phe	994	HMDB0000159	301	288	334	314	310	288	307	24	304	14	1.0	0.851	
A_0027	Phosphoenolpyruvic acid	1005	HMDB0000263	13	14	12	13	20	21	13	1.0	18	4.3	0.7	0.186	
C_0034	Pro	614	HMDB0000162,HMDB0003411	306	259	343	305	309	299	303	42	304	5.4	1.0	0.953	
A_0099	PRPP	7339	HMDB0000280	N.D.	N.D.	N.D.	N.D.	5.9	N.D.	N.A.	N.A.	5.9	N.A.	<1	N.A.	
C_0009	Putrescine	1045	HMDB0001414	4.2	5.5	3.8	5.5	5.0	3.1	4.5	0.9	4.5	1.3	1.0	0.961	
A_0003	Pyruvic acid	1060	HMDB0000243	N.D.	N.D.	N.D.	136	147	121	N.A.	N.A.	135	13	<1	N.A.	
A_0065	Ribose 5-phosphate	439167	HMDB0001548	167	127	102	69	97	56	132	33	74	21	1.8	0.071	
A_0064	Ribulose 5-phosphate	439184	HMDB0000618	707	667	661	574	613	590	678	25	592	20	1.1	0.011	*
C_0193	*S*-Adenosylmethionine	34755	HMDB0001185	56	56	64	29	37	35	58	4.7	34	4.1	1.7	0.003	**
C_0011	Sarcosine	1088	HMDB0000271	32	23	33	51	32	34	29	5.5	39	10	0.7	0.236	
A_0079	Sedoheptulose 7-phosphate	165007	HMDB0001068	281	95	77	187	225	203	151	113	205	19	0.7	0.498	
C_0026	Ser	617	HMDB0000187,HMDB0003406	735	694	719	578	630	657	716	21	622	40	1.2	0.037	*
C_0085	Spermidine	1102	HMDB0001257	36	41	26	31	31	30	34	7.9	31	0.5	1.1	0.511	
C_0133	Spermine	1103	HMDB0001256	8.8	7.8	7.6	13	14	13	8.1	0.6	14	0.3	0.6	6.1E-04	***
A_0010	Succinic acid	1110	HMDB0000254	535	721	724	482	623	475	660	108	526	84	1.3	0.170	
C_0041	Thr	6288	HMDB0000167	493	469	514	437	453	451	492	22	447	8.5	1.1	0.059	
C_0151	Thymidine	5789	HMDB0000273	3.0	2.2	2.4	N.D.	2.2	N.D.	2.5	0.4	2.2	N.A.	1.1	N.A.	
C_0054	Thymine	1135	HMDB0000262	N.D.	N.D.	N.D.	N.D.	N.D.	N.D.	N.A.	N.A.	N.A.	N.A.	N.A.	N.A.	
C_0136	Trp	1148	HMDB0000929	72	68	81	61	64	56	74	6.8	60	4.0	1.2	0.054	
C_0118	Tyr	1153	HMDB0000158	254	223	281	237	217	184	253	29	212	27	1.2	0.153	
C_0076	Tyramine	5610	HMDB0000306	N.D.	N.D.	N.D.	N.D.	N.D.	N.D.	N.A.	N.A.	N.A.	N.A.	N.A.	N.A.	
A_0103	UDP	6031	HMDB0000295	6.9	6.9	5.7	7.7	6.4	7.1	6.5	0.7	7.0	0.6	0.9	0.392	
A_0086	UMP	6030	HMDB0000288	131	118	141	175	145	57	130	11	126	61	1.0	0.913	
C_0031	Uracil	1174	HMDB0000300	61	63	67	57	69	90	63	3.0	72	17	0.9	0.473	
C_0153	Uridine	6029	HMDB0000296	460	368	384	192	221	283	404	49	232	46	1.7	0.012	*
A_0116	UTP	6133	HMDB0000285	N.D.	N.D.	N.D.	N.D.	N.D.	N.D.	N.A.	N.A.	N.A.	N.A.	N.A.	N.A.	
C_0036	Val	1182	HMDB0000883	488	479	529	545	535	456	499	26	512	49	1.0	0.701	
C_0010	β-Ala	239	HMDB0000056	123	175	205	279	279	275	167	42	278	2.6	0.6	0.044	*

N.A., Not Available; N.D., Not Detected

¶ The ratio of the detected mean values between the two groups.

‖ Welch's t-test (*p<0.05, **p<0.01, ***p<0.001)
